# Developing an Evidence-Based Technical Assistance Model: a Process Evaluation of the National Training and Technical Assistance Center for Child, Youth, and Family Mental Health

**DOI:** 10.1007/s11414-020-09686-5

**Published:** 2020-01-23

**Authors:** Jonathan R. Olson, Jennifer Schurer Coldiron, Ryan M. Parigoris, Michelle D. Zabel, Marlene Matarese, Eric J. Bruns

**Affiliations:** 1 Department of Psychiatry and Behavioral Sciences, University of Washington, Building 29, 6200 NE 74th Street, Suite 100, Seattle, WA 98115 USA; 2 Department of Community and Human Services-King County, Chinook Building, 401 5th Ave Ste 500, Seattle, WA 98104 USA; 3 Department of Clinical Psychology, 100 William T. Morrissey Blvd, Boston, MA 02125 USA; 4 The Institute for Innovation & Implementation, University of Maryland School of Social Work, 306 West Redwood Street, Baltimore, MD 21201 USA

## Abstract

The National Training and Technical Assistance Center for Child, Youth, and Family Mental Health (NTTAC) supports the development and implementation of systems of care (SOC) for youth with serious emotional disorders (SED) and their families. This article presents results from a process evaluation of NTTAC, conducted to support the Center’s quality improvement and contribute to the knowledge base around provision of technical assistance (TA). The evaluation used a mixed methods approach with data collection focused on a defined subset of NTTAC TA recipients—recipients of federal Comprehensive Community Mental Health Services for Children SOC grants. Data sources included coded administrative records from SOC grant sites, administrative data from NTTAC, standardized measures of SOC development, and stakeholder survey data. Results indicate that TA dosage matched needs and goals of TA recipients (SOC sites), overall levels of satisfaction with TA were high, and TA content was generally aligned with need. TA recipients reported significant progress on indicators of SOC development over time. Together, these findings suggest that it is possible to develop TA methods that reflect the level and type of TA recipients’ goals and needs, and, in turn, positively impact SOC development and behavioral health service delivery.

The field of children’s mental health has long recognized shortcomings in child-serving systems’ capacity to meet the needs of youth with serious emotional and behavioral disorders.^1, 2^ Among the many challenges associated with serving this population, a primary factor is inadequate coordination among the multiple systems, services, and supports necessary to help these youth and their families. One response to this challenge has been to implement systems of care (SOC)—a philosophy that provides guidance for helping youth and families access and navigate through a continuum of services and supports that are individualized, based on individual family needs, driven by family priorities, and built upon family strengths.^3, 4^ Over the past two decades, the SOC framework has been widely adopted by states, regions, and localities as a strategy to coordinate care for youth with the most serious behavioral health needs. However, given the complexities associated with coordinating multiple systems, those implementing SOC practices have needed assistance to support implementation efforts.^4^ Such need has resulted in the creation of several technical assistance (TA) systems that have been designed to help support SOC implementation efforts.

In recent years, TA provision has become a common component of the implementation process for a wide variety of youth-focused federal, state- and community-based initiatives spawning a small but growing body of literature focused on evaluating and identifying characteristics of effective TA.^5–10^ There has been growing interest in studying how to use training and TA to support implementation of specific evidence-based practices, and also an emerging dialog about how best to use TA to improve the overall functioning of larger organizations and systems. Moreover, there has been consistent interest in ensuring that TA systems themselves are based on comparative effectiveness research.^6, 9, 11^ However, empirical research on the effects of TA on general youth-focused programming is emergent at best, with significant gaps in research on the provision of TA to support the implementation of SOC strategies.

The purpose of this paper is to contribute to the nascent literature on what constitutes “evidence-based TA,” particularly as applied to support the implementation of systems of care for youth and their families. The paper presents findings from a process evaluation of one aspect of the efforts of the National Training and Technical Assistance Center for Child, Youth, and Family Mental Health (NTTAC); specifically, TA provided by NTTAC to states, tribes, territories, and communities that received federal funding from the Substance Abuse and Mental Health Services Administration (SAMHSA) to reform and improve systems of care for children, youth, and young adults with serious emotional disorders (SED) and their families. The introduction will focus on the theory and research base on provision of effective TA in health and human services delivery, which provided guidance to both NTTAC’s activities and the current research study. It will also describe NTTAC, its mission and activities, and conclude by describing the aims of the current evaluation research project.

## Technical Assistance Defined

Technical assistance has been defined as an individualized approach that provides implementation support to, and increasing capacity for, continuous quality improvement (CQI) among program developers, service providers, managers, and decision makers.^8, 12^ Technical assistance providers typically have specialized knowledge and experience that is relevant to a particular implementation effort, and they use their skills to help build and improve services and systems. In practice, TA encompasses a wide variety of strategies including training, coaching, educating, problem-solving, and generally supporting relevant stakeholders.^13–15^

Le and colleagues have suggested two general categories of TA: content-driven and relationship-based.^9^ Content-driven TA typically involves sharing information, making referrals, and relying on data-based resources. Relationship-based TA focuses on building partnerships among TA providers and various stakeholder groups with the goal of promoting positive and productive changes in individuals, organizations, and systems that support implementation efforts. Scholars have further categorized TA by level of intensity. Basic, or generalized, TA focuses on raising awareness through education and support. Individualized TA is tailored to meet the unique needs of individual stakeholder groups, and intensive TA promotes new knowledge through concentrated efforts aimed at supporting organization- and system-level changes.^16, 17^

### Characteristics of High-quality TA

In recent years, a growing body of literature has begun to elucidate specific characteristics, or core components, of effective TA. For example, Wandersman and colleagues underscore the importance of four dimensions of TA.^8^ First, TA must be of sufficient dosage. Recent evidence suggests that long-term, ongoing TA is associated with better outcomes than one-shot, time-limited efforts.^6, 9, 10^

Second, TA should encompass a mixture of intensive support (e.g., on-site consultation and engagement in in-person and virtual learning communities) that provides opportunities for experiential learning through hands-on demonstrations and off-site check-ins via telephone and electronic communications that allow for cost-efficient, frequent contacts.^15, 18, 19^ Results of recent studies have underscored the importance of providing individualized and tailored feedback to help TA recipients translate general support into concrete action steps.^15, 18, 19^ Meanwhile, peer-to-peer approaches to TA provision such as learning communities have been found to be associated with implementation fidelity and sustainability of evidence-based practices over time.^20^

Third, TA activities benefit from a collaborative approach in which TA providers offer expert guidance in specific substantive areas, and also form working relationships with various stakeholders, including policy-makers, program administrators, implementers, and service recipients.^15^ Several scholars have suggested that such collaborative efforts build trust, positive communication, and empathy—all of which help build partnerships between TA providers and recipients.^9, 13, 15^

Finally, recent papers have suggested that there are benefits associated with a proactive approach to TA, in which TA providers anticipate stakeholder needs and respond to those needs in a timely, positive, and ongoing way.^8, 21^ Proactive TA typically builds upon stakeholder strengths with the ultimate goal of improved systems-level capacity and high-quality implementation.^7^

### Phases of TA Implementation

A growing body of literature has focused on identifying specific stages or phases of technical assistance, in which TA activities vary according to the developmental period of a particular effort. Le and colleagues have suggested that successful TA starts with a decision-making process in which TA needs are identified, and TA plans are developed in a way that is consistent with stakeholder needs, values, and currently available resources.^9^ Similarly, Wandersman and colleagues have suggested that during the early stages of implementing a new TA effort, steps must be taken to identify ways to build upon the strengths of TA recipients, to identify specific goals and expected outcomes, and ensure that TA activities fit with recipients’ circumstances.^8, 21^ In recent years, there has been movement towards data-driven TA, in which relevant data points are identified and reviewed to guide TA efforts.^22, 23^ Such data-driven approaches help ensure that TA is delivered to those who need it, not only to those who make a formal request for support. Furthermore, such a systematic approach helps TA providers tailor their support to the unique goals, strengths, and needs of recipients.

Following the decision-making phase, specific strategies are then developed and implemented. According to the results of their qualitative interviews with TA providers, Le and colleagues suggest that this process is most successful when TA plans are flexible and individualized, build upon current skills and competencies, and strengthen collaborative relationships.^9^ Others have echoed these ideas as they have called for a collaborative approach to TA implementation in which the specific roles of TA providers are well-defined and complement the needs, current resources, and capacities of TA recipients that were defined during the decision-making phase.^7, 21^

There seems to be general agreement that an important third phase of the TA process is to build in efforts to evaluate the impact of TA activities. Le et al. underscore the importance of evaluating stakeholder perceptions of adequacy of and satisfaction with the TA received.^9^ Similarly, Katz and Wandersman have suggested that process and outcome evaluations should be used to assess TA quality, reach, dosage, and recipient satisfaction, and that stakeholders should also plan for longer term outcome evaluations.^21^ Lessons learned from these efforts can then be used to improve TA efforts as a part of a general continuous quality improvement plan. However, the process of creating an actual field of study related to evidence-based technical assistance remains emergent.

## National Training and Technical Assistance Center for Child, Youth, and Family Mental Health

The focus of the current study is to evaluate efforts of the National Training and Technical Assistance Center for Child, Youth, and Family Mental Health (NTTAC) for Child, Youth, and Family Mental Health. The Center is part of the Technical Assistance Network for Children’s Behavioral Health (TA Network), hosted by the University of Maryland School of Social Work. The primary mission of NTTAC is to provide TA related to children’s behavioral health to states, tribes, territories, and communities. Although NTTAC is responsible for providing TA to any state, tribe, territory, or jurisdiction that requests support, the focus of this evaluation is on TA provided to recipients of Comprehensive Community Mental Health Services for Children and Their Families Initiative (CMHI) grants, which are funded through Substance Abuse and Mental Health Services Administration (SAMHSA).

NTTAC is composed of a group of partner organizations that are national and international content experts in the area of children’s behavioral health, as well as over 50 expert individual consultants. NTTAC also includes the National Indian Child Welfare Association (NICWA), which is primarily responsible for providing TA to Tribal CMHI systems of care (SOC) grantees, but operates as a core partner of NTTAC using similar strategies. Core features of the TA provided by NTTAC include supporting adoption of the principles and operating procedures of the SOC approach. The ultimate goal is to promote systems-level conditions that support the implementation of a comprehensive array of evidence-based psychosocial interventions and other supports for children and youth with or at risk of emotional and behavioral disorder, in order to help them to function better at home, in school, and in the community.^8, 21, 24^

### TA Strategies Employed by NTTAC

NTTAC adheres to many of the theory- and research-based techniques reviewed above in its work with state, county, local, and tribal systems of care. Specifically, the Network delivers content-driven and relationship-based proactive TA that is tailored to the unique needs of CMHI grantees. The Network provides basic generalized TA in the form of weekly communications with grantees; regular updates and webinars; convening of Learning Communities on topics of particular interest to the field; and general content-specific publications, products, and resources. In addition, grantees receive individualized TA by being matched with expert consultants who provide tailored support to their projects’ goals and system development needs.

Intensive TA occurs through regular communication via telephone and electronic technologies, on-site visits, and connection to peers in the field (e.g., representatives from other states and/or CMHI grantees) from whom they may glean information about successful strategies. TA providers encourage CQI and introduce grantees to supportive measures and tools designed to track and assess implementation. They also share resources that help keep grantees current on new innovations in areas such as intensive care coordination models, crisis response, managed care, family- and youth-provided peer-to-peer support, evidence-based psychosocial interventions, and policy and financing mechanisms that can support these innovations (see https://theinstitute.umaryland.edu/our-work/national/network/about/).

NTTAC works with CMHI grantees throughout their grant cycles from program planning through the stages of implementation, evaluation, and sustainability. The general TA approach is flexible, but largely follows the steps outlined above. For example, NTTAC TA consultants assess grantees’ current implementation processes and adjust support to meet the unique needs of each stakeholder. In line with the recommendations of Wandersman and colleagues and Le and colleagues, TA topics vary across sites and delivery mechanisms are flexible.^8, 9^ Examples of NTTAC priorities include designing and implementing effective organizational structures, developing sustainable financing for services and systems, family- and youth-driven care, promoting clinical best practices, implementing CQI, and coordinating cross-system work to ensure that the diverse and complex needs of youth and their families are best met.

## Effects of Technical Assistance on Implementation

Le and colleagues have noted a relative dearth of high-quality data focused on the effectiveness of TA.^9^ However, a small but growing body of literature is beginning to emerge. At the systems level, Kahn and colleagues reported preliminary results from a summative evaluation of TA efforts aimed at promoting sustainable systems change in early intervention and preschool special education programs.^25^ Specifically, implementation of the TA model for long-term systems change was associated with improved state systems, including creation of new agreements across agencies, improved monitoring systems, and refined data systems to help guide state decision-making. TA efforts also were associated with improved local infrastructure, including increased service coordination and streamlined policies and structures that support eligibility determination and assessments. At the inner setting level, TA was associated with improved practitioner skills and knowledge, and at the family level, TA was linked with better access to services. The researchers found that such outcomes were more likely under conditions of balanced leadership with TA providers taking the lead on plan development but not “owning” the plan. In addition, systems-level outcomes were associated with following an established written implementation guide and dedicating sufficient time to plan development.

Many studies have focused exclusively on the impact of TA on administrative and staff-related outcomes within organization and/or provider-levels. For example, several research teams have found TA to be linked with positive implementation team functioning and enhanced staff skills.^5, 26^ Bryson and Ostmeyer found that a combination of focused trainings and follow-up coaching sessions were associated with enhanced staff skills in implementing programs for children with autism spectrum disorder.^26^

Recent studies have suggested that the impact of TA is at least partially dependent on the degree to which specific support is both timely and tailored to the unique needs of stakeholders. Chilenski and colleagues found that levels of collaboration among TA providers and prevention team members during one phase of program implementation were associated with more positive team functioning in later phases.^5^ A related study focused on collaborative relationships among TA providers and community prevention teams over time. Chilenski and colleagues found that levels of collaboration were relatively high during the planning phase of a community prevention project, decreased during implementation, and then increased again during the sustainability phase.^11^ Such findings underscore the importance of tailoring TA to the unique needs of stakeholders that typically change throughout different phases of planning and implementation.

Other studies have focused on the impact of TA dosage on specific implementation outcomes at the program, rather than system or organizational levels. For example, several studies have suggested that higher doses of TA are associated with an increased likelihood that a strategy has been implemented successfully or a specific program has been implemented with fidelity to its underlying model.^6, 15, 16^ The relation between dosage and outcomes appears; however, to be moderated by level of TA need such that dosage has less influence on outcomes when specific implementation tasks are either very easy or very difficult. For example, easy tasks might be accomplished even with minimal TA support, while difficult tasks may be slow to develop even with high levels of TA support.^6, 12, 16, 18, 27, 28^ Furthermore, studies on this topic suggest that examining simple linear relations between TA dosage and outcomes does not capture the complexity of the relation between these two variables. For example, Feinberg and colleagues found a positive association between TA dosage and community prevention board functioning.^6^ However, such effects were limited to only newly formed boards. Such findings underscore the importance of focusing TA efforts to fit with the contextual and systems-level factors that will influence their ultimate effectiveness.

### Expanding the Research Base

In light of the limited number of studies that have focused on evaluations of TA, several scholars have outlined priorities for future research. For example, Le and colleagues have underscored the importance of concentrating on the implementation stage of the TA process and identifying the extent to which action steps have been implemented by stakeholders.^9^ They suggest that studies could focus on policy, systems, and service delivery levels. Katz and Wandersman have suggested a need for studies that elucidate the degree to which TA may vary across different types of situations in real-world settings, stressing the importance of considering context in TA delivery.^21^

## The Current Study

The purpose of this study was to conduct a process evaluation of NTTAC with a focus on outcomes related to both the decision-making and implementation phases of TA.^9^ Within the *decision-making* phase, the study focused on identifying stakeholder needs, and measuring the overall reach of the TA efforts. Within the *implementation phase*, the study focused on stakeholder satisfaction with TA activities, alignment of TA activities with stakeholder needs, and short-term systems-level outcomes related to select implementation activities. Specific research questions were:

1. What are the TA needs of stakeholders as reflected in CMHI grantees’ project goals?
2. What is the “reach” of NTTAC in terms of amount of TA provided across state, county, territory, and tribal jurisdictions?
3. How satisfied are TA recipients with NTTAC assistance and products?
4. To what degree does the TA provided by NTTAC align with needs of grantees?
5. To what degree did recipients (funded CMHI sites) of TA from NTTAC successfully implement the SOC framework, in areas such as governance, management policies and procedures, support of local service delivery, and CQI?

## Methods

### Participants

Participants in this study included recipients of CMHI SOC grants, which were funded through SAMHSA. Thirty five CMHI grantees received funding in 2013/2014 and another 67 grantees received funding between 2015 and 2017. Grantees are diverse in location, size, region, jurisdiction, and award amount. Table 1 provides a summary of their characteristics. Descriptions of grant jurisdiction differed across cohorts. In order to explore differences by type of project and grantee catchment area, grantees were divided into three categories: local (comprising “local” and “county” grantees), state (comprising “state,” “territory,” and “regional” grantees), and tribal (see Table 1). Across these categories, 41 states and 3 territories are represented in the sample, with number of grants per state ranging from 1 to 7.

**Table 1:** Characteristics of CMHI grantees

Cohort	Number of grantees	Mean (range)	Mean (range) year of first grant	Grant jurisdiction		
		Grant amount		Number of county/local	Number of state/territory	Number of tribal
2013	14	$924,124	2004	2	8	4
		($328,744–$1.00 M)	(1984–2013)			
2014	21	$1,810,000	2010	8	8	5
		($447,851–$4.00 M)	(1998–2014)			
2015	24	$1,986,218	2008	20	3	1
		($573,410–$12.00 M)	(1996–2014)			
2016	32	$1,605,599	2007	17	11	4
		($783,468–$3.00 M)	(1992–2016)			
2017	11	$1,908,365	2011	2	8	1
		($744,236–$3.00 M)	(1999–2017)			
2013/14 Cohort	35	$1,454,822	2008	10	16	9
		($328,744–$4.00M)	(1984–2014)			
2015/16 Cohort	67	$1,791,648	2008	39	22	6
		($573,410–$12.00M)	(1992–2017)			
All	102	$1,676,071	2008	49	38	15
		($328,744–$12.00M)	(1984–2017)			

### Measures

#### Grantee Summary Reports

Grantee sites’ goals were coded from reports provided by the national evaluation of the CMHI program that summarized elements of each grantee’s proposal and SOC development plan.^29^ These reports were based on each site’s CMHI grant application, interviews, and other interactions with each grant recipient’s key stakeholders. These summary reports included grantee descriptors, such as grant goals, award size, TA provider, award number, and grant type.

#### TA Topics and TA Dosage

NTTAC providers log TA activities in the Technical Assistance Reporting System (TARS). TARS has been in operation since July 2014, and the system helps NTTAC track details about each TA contact, such as the recipient, provider, date, length, format of the contact, and the topics covered. There is also a narrative section that briefly summarizes the content of the contact and next steps. For the current study, all TARS contact entries through March 31, 2018 were downloaded from TARS for each CMHI grantee across both cohorts. This totaled 3926 entries (2133 for the 2013/14 cohort, and 1793 for the 2015–17 cohort). These files were then merged into a master database of all contacts and eventually aggregated by grantee and merged with other data from sources described below.

Before aggregation, the topics covered in each contact were recoded into descriptive and discrete categories. Throughout the history of TARS use by NTTAC, the topics “pick list” has undergone several iterations. For example, in order to simplify and streamline the TARS system, a change of topics was implemented on April 1, 2018, eliminating “EBPs” as a selectable topic. In order to meet the current project’s aims and have consistent topic coding across TARS topics from multiple iterations of the platform as well as from the multiple data, the 143 possible TARS topics were collapsed into 19 overarching topical themes. The development of these new topic categories was based on logical groupings of TARS themes, as well as topics that emerged while coding other data sources.

#### Grantee Satisfaction with TA

Since April of 2016, NTTAC has administered a quarterly online quality improvement measure (QIM) to grantees that received individualized TA from NTTAC affiliates in order to monitor recipients’ satisfaction with and the impact of TA provided. TA recipients are identified via TA providers’ logs of TA contacts into TARS. The QIM gathers basic information about the TA recipient, and then asks each recipient to report which topics they received TA; how much impact the TA had on the quality of mental health programs and services for children, youth, and families at their site; and the helpfulness of each TA provider they interacted with in the past quarter. Self-reported satisfaction with individualized forms of TA was operationalized as a rating of overall helpfulness in which participants responded to individual items within an index with response categories ranging from a low score of 1 for “unhelpful—made things worse” to a high score of 5 for “extremely helpful.” Self-reported satisfaction with generalized forms of TA was rated on a 10-point index, with potential ratings ranging from 0 for “very unsatisfied” to 10 for “very satisfied.” Similarly, reports of impact of TA on the quality of programming were rated on an item with response categories ranging from a low score of 0 for “none” to a high score of 10 for “profound and enduring.” Response rates for these surveys ranged from 25 to 30%, with about 45–60 respondents completing surveys each quarter.

#### Self-Assessment of Implementation Survey

The Self-Assessment of Implementation Survey (SAIS) is a multi-informant online survey administered as part of the CMHI national evaluation to administrators involved in implementing CMHI grant goals. The purpose of the SAIS is to assess the degree to which strategies have been implemented in the service of developing or expanding the grantee’s system of care. The SAIS focuses on inner and outer setting activities at the jurisdiction (state, county, or tribal) level. Given the focus of this instrument on implementation outcomes, it was appropriate for use in the current analyses. There are five major themes of the SAIS, each evaluated via a series of self-report questions:

1. *SOC Governance:* items related to the policies, procedures, and routines of the jurisdiction-level governing body/structure overseeing the SOC implementation/expansion.
2. *Management, policies, and procedures:* items related to management, policies, and procedures that impact operations and service delivery. This section also assesses strategies to ensure that youth and families have access to a comprehensive array of community-based services and supports that can promote positive emotional/behavioral outcomes and maintain youths in their homes and communities.
3. *Support of local service delivery:* items related to the availability of key services within the jurisdiction (i.e., service array adequacy) and what strategies have been implemented to support the use of evidence-supported interventions, utilize person-centered, outcomes-based planning modalities and service coordination, and reach underserved populations.
4. *Geographic area covered:* items related to representation of relevant system partners and other stakeholders in SOC governance activities and what strategies are used to share information with such partners, provider agencies, and others.
5. *Continuous quality improvement:* items related to accountability routines and data collection and use.

The SAIS presents a list of relevant strategies for each of the above themes and asks respondents to choose a response between 1 for “not planned” and 6 for “extensively implemented” that best describes the level of development of each strategy. Mean scores for each of the themes were calculated, along with mean scores for all of the items across each theme.

Over the course of 2016, the SAIS was administered to between five and 13 people at each of the 35 2013/2014 CMHI grantee sites (a total of 280 people). A total of 159 people responded for an overall response rate of 57%, with individual response rates per site ranging from 12.5 to 100% (mean = 58.1%). Some sites had data available from as many as eight respondents and others had data available from only one respondent (mean = 5.0 respondents). Responses were aggregated by grantee, with the mean score and standard deviation of each item and theme score retained for use in analyses.

Although the SAIS provides a potential measure of longitudinal SOC development progress across time and CMHI cohorts, resource limitations restricted the use of the SAIS by the national evaluation team to periodic, cross-sectional assessment. Beginning in 2018, a follow-up SAIS was administered to select CMHI grantees from the 2014 cohort since they still received funding during 2018. Thus, analyses were limited to baseline and follow-up data collected from this cohort. Similar to the first wave of data collection, the SAIS was re-administered to between six and 10 people at 18 of the 2014 grantee sites (a total of 146 people). Responses were submitted by 107 respondents, for a total response rate of 73%. The number of respondents for a given site ranged from three to eight with a mean of 5.94 respondents. Respondent IDs were not tracked for either administration of the SAIS, so the degree to which individual respondents from the initial SAIS were the same respondents completing the follow-up is unknown, but responses were aggregated by grantee, giving the ability to track changes at the system, rather than individual level.

### Analytic Strategy

#### TA Needs of Stakeholders

TA needs of stakeholders were identified by coding goals from the grantee summary reports. Two coders coded grants independently based on key terms associated with specific TA needs, as expressed in grant goals. For the 2013–2014 cohorts, coders established inter-rater reliability on a sample of grants and then coded the remainder independently. Specifically, two research assistants coded the TA needs of stakeholders for each site based on a coding scheme related to key words for each identified need. Any discrepancies were discussed in-person until an agreed upon response was determined. Therefore, although there was 100% agreement between the coders, there was no objective measure of inter-rater reliability. A similar procedure was used for the 2015–2017 cohorts, where coders coded all grants independently and then met to discuss discrepancies and reach agreement. The research team used the coded goals as a proxy for TA needs and summarized those using simple percentages for each cohort of grantees.

#### Reach of NTTAC

TA reach was assessed by computing simple mean hours of TA, and categorizing users into low-, medium-, and high-usage levels. Comparisons were then made across type of jurisdiction: county, state/territory, and tribal. Differences in levels of usage across these jurisdictions were assessed using chi square analyses. In addition, associations between TA usage and levels of SOC development were assessed by using One-way Analysis of Variance (ANOVAs) to compare differences in mean SAIS subscale scores across low, medium, and high levels of TA usage. Tukey’s honestly significant difference (HSD) post hoc test was used to identify significant differences across levels of TA usage.

#### Grantee Satisfaction with TA

Mean satisfaction and perceived impact scores were computed separately for five quarters, from fiscal year 2017, quarter three, through fiscal year 2018, quarter three.

#### Alignment of TA with Stakeholder Needs

The degree of alignment of stakeholder needs with the actual TA provided was assessed by running associations between the number of grantees with a particular topical goal against the number of grantees that received TA about that topic. TA topics were based on stated grant goals, and topics covered were identified through TARS data. Differences between topical goals and TA topics actually covered were assessed through a series of chi square goodness of fit tests.

#### Impact of TA on Program Implementation

Changes in implementation activities were measured using repeated-measures *t* tests to assess changes in SAIS subscale scores from 2016 to 2018.

## Results

### Grantees’ Initial Goals as Stated in Their Summary Reports

Table 2 presents the results of the coding of all grantees’ (2013–2017; *N* = 102) SOC implementation or expansion goals from each grantee’s summary report. While grantees’ goals varied widely, all participants (100%) reported goals in the areas of building SOC infrastructure and enhancing governance and collaboration. The majority of grantees also had goals in the areas of developing an accessible service array, development of the mental health workforce, and family involvement and leadership.

**Table 2:** Topics used by TA providers

Grant goal topics	2013–14 Cohort		2015–16 Cohort		All grantees	
	*n*	%	*n*	%	*n*	%
SOC infrastructure, governance and collab	35	100%	67	100%	102	100%
Service array and access	35	100%	60	90%	95	93%
Workforce development	31	89%	58	87%	88	86%
Family involvement and leadership	29	83%	54	81%	83	81%
Cultural and linguistic competence	31	89%	51	76%	81	79%
Communications and advocacy	27	77%	52	78%	79	77%
Youth involvement and leadership	29	83%	48	72%	77	75%
Financing strategies	28	80%	47	70%	75	74%
Wraparound and other CC approaches	24	69%	51	76%	75	74%
Evaluation, CQI, and research	20	57%	49	73%	69	68%
EBPs	22	62%	47	70%	68	67%
Trauma-informed services/systems	23	66%	41	61%	64	63%
Peer support (youth or family)	12	34%	45	67%	57	56%
Referrals, screening, and eligibility	9	26%	38	57%	47	46%
Technology	10	29%	19	28%	29	28%
Rural considerations	11	31%	9	13%	20	20%
Tribal considerations	8	23%	7	10%	16	16%

### Reach of NTTAC

For grantees whose awards began in late September of 2014, TA utilization varied dramatically by grantee. Table 3 depicts the mean number of hours of TA 2014 grantees received through July of 2017, with TA usage categorized into high/medium/low based on natural breaks in the sum of hours of TA received. TARS data after this date were excluded from this section in order to best represent TA received up to the time of the first SAIS administration. As shown, tribal grantees are more likely to be high users of TA as compared with state and county grantees (*χ*^2^ = 12.85, *p* = 0.007). Additionally, high users of TA reported significantly less progress than low users of TA in the areas of governance (*F* = 4.22, *p* = 0.032), management policies and procedures (*F* = 5.11, *p* = 0.018), and geographical area covered (*F* = 5.77, *p* = 0.012). Tukey’s HSD tests indicate that the significant differences were between high- and low-level users of TA with no significant differences found for medium-level users. There also were no significant differences across TA levels for progress on supporting local service delivery (*F* = 0.74, *p* = 0.492) or continuous quality improvement (*F* = 1.90, *p* = 0.183).

**Table 3:** Characteristics of high and low TA utilizers in 2014 grantee cohort

TA usage category	*n*	Mean/range TA hours received	Chi square analyses: TA usage by grant jurisdictions			ANOVA analyses: mean SAIS rating scores across TA usage categories				
			Local	State	Tribal^+^	Governance*	Management*	Local delivery	Geographic area covered*	Continuous quality improvement
Low	8	24.9	5	3	0	3.98*	3.82*	4.10	4.28*	3.46
		(7–53.2)								
Medium	8	82.4	3	4	1	3.85	3.69	3.84	3.72	3.35
		(68.1–107.5)								
High	5	242.54	0	1	4	3.09*	2.97*	3.63	3.20*	2.56
		(162.8–322.3)								
All 2014 grantees	21	95.9	8	8	5	3.75	3.6	3.91	3.84	3.27
		(7–322.3)								

^+^*p* < 0.05 with tribal grantees more likely to be high users of TA than local or state grantees (based on chi square analyses)

*p < 0.05 with statistically significant differences between low and high users of TA (based on ANOVA and Tukey post hoc)

### TA Recipient Satisfaction

Across all respondents and time points assessed, ratings of satisfaction and perceived impact of NTTAC’s assistance and products were very high. Satisfaction with individual TA providers was rated the highest, at an average of 4.72 out of a possible 5 points. Respondents rated their satisfaction with assistance and products provided by NTTAC at 7.56 on a scale of 0 (None) to 10 (High). When asked on a scale from 0 (None) to 10 (Profound and Enduring), *How much impact do you think your interactions with the TA Network/NICWA in the last 3 months will have on the quality of the mental health programs and services for children, youth, and families in the site you are a part of?*, TA recipients rated the TA’s impact at mean scores of at least 7 out of 10.

### Alignment Between TA Provided and Grantee Needs

**Fig. 1:**
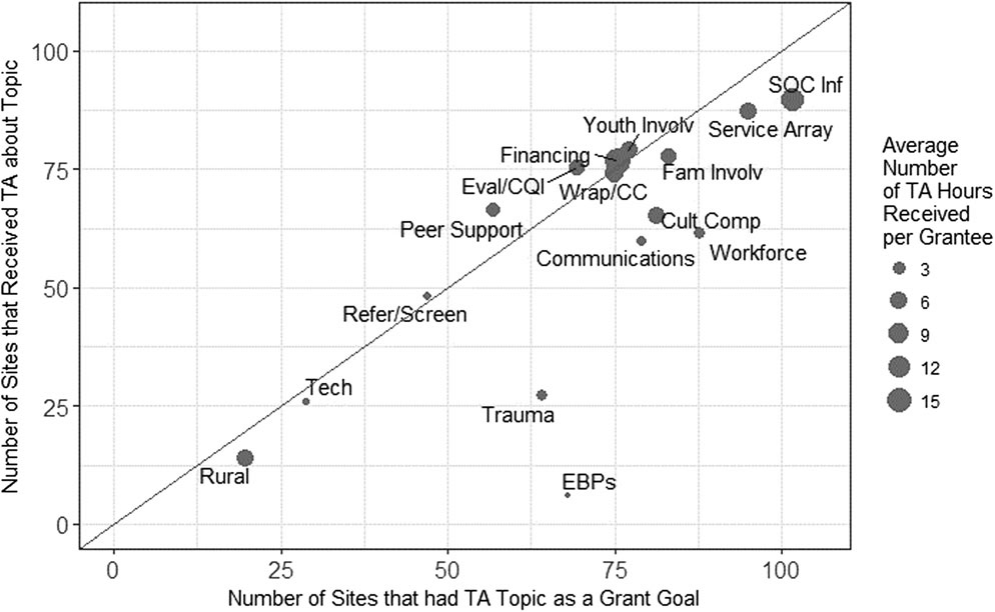
Number of grantee sites with specific goals vs. number that received ta on those goals (all grantees; *N* = 102)*

Based on TARS entries, the number of sites with each TA topic-specific goal outlined in Table 2 was linked with the exact amount of TA received on each topic. Figure 1 provides a visual depiction of the degree of alignment between sites’ stated goals and TA actually received. Topics of “tribal considerations” and “other” were excluded from the graph since “tribal considerations” was not a selectable topic in TARS and goals were not coded on “other.”

A series of chi square goodness of fit tests was run to examine if there were statistically significant differences between types of TA that were expected based on grant goals, and those that were actually received by each grantee as indicated in the TARS database. As shown, nine of the sixteen topics have an almost perfect match of the number of sites potentially needing and then receiving TA on a particular topic (see Fig. 1). Results of the chi square analyses indicate that grantees received significantly less TA than expected on the following topics: trauma-informed services/systems (*χ*^2^ = 55.87, *p* < 0.001), evidence-based practices (*χ*^2^ = 172.79, *p* < 0.001), communications (*χ*^2^ = 18.96, *p* < 0.001), cultural competence (*χ*^2^ = 16.50, *p* < 0.001), workforce development (*χ*^2^ = 65.94, *p* < 0.001), service array and access (*χ*^2^ = 7.62, *p* = 0.006), and system of care infrastructure and governance (*χ*^2^ = 101.00, *p* < 0.001).

### Impact of TA on Implementation

**Fig. 2:**
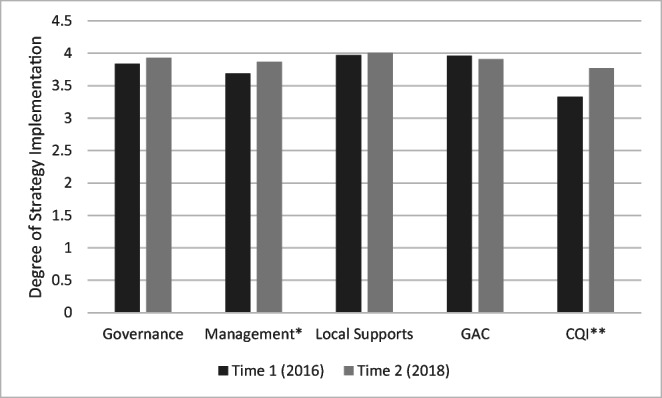
Change in self-reported strategy implementation over time, 2014 CMHI Grantees (*N* = 18). ^**^Denotes a statistically significant difference in repeated-measures t-tests (*p* < 0.05). ^*^Denotes a difference trending towards significance in repeated-measures *t* tests (*p* < 0.10)

Figure 2 displays mean scores across the five SAIS domains for the 2016 and 2018 administrations. Results of paired samples *t* tests indicate that grantees reported a significant increase in CQI (operationalized as accountability routines, and data collection and use) over time (*t* = 2.91, *p* = 0.01). Furthermore, the management domain increased over time, with the result trending toward but not reaching statistical significance (*t* = 1.93, *p* = 0.07). The three other subscales remained relatively unchanged.

## Discussion

The results of this study contribute to the small but growing body of literature aimed at explicating methods for undertaking data-informed TA efforts while also providing actionable information for use by NTTAC and the TA Network in general. Results indicate that CMHI SOC grantee sites supported by NTTAC reported a wide variety of goals related to building strategies and systems in the inner and outer settings that increase capacity to better meet the behavioral health needs of children and youth within states, jurisdictions, territories, and tribes. Furthermore, NTTAC has had a broad reach, although there were variations in TA provided across levels of jurisdiction that may point to needed improvements in partnership and TA provision.

For example, counties tend to be rather low users of TA, compared with states and tribal grantees. This may be a function of the relatively greater availability of individuals in state systems to lead funded CMHI grant efforts than in county or local systems. NTTAC may need to allocate additional time to developing relationships with leaders in county and local grantee sites, to ensure their TA needs are being met. Overall, however, findings related to TA dosage suggest that the level of TA contact may be well-aligned with the level of sites’ needs, given that results indicate that sites struggling with implementation are receiving more TA than those that have made more progress with implementation. Such findings are consistent with previous studies that have suggested that the relationship between TA dosage and outcomes is not necessarily linear. For example, Chinman and colleagues found strong positive correlations between TA hours and program performance when TA recipients were focused on complex tasks that benefited from high levels of TA attention; but that the relation between TA dosage and outcomes was less clear for tasks at different levels of scope and difficulty.^12^ Similarly, Feinberg and colleagues found that TA dosage was positively associated with community prevention board functioning, but only early in the implementation process when these boards were initially forming.^6^ The findings from the current study add to this growing understanding of the contexts in which TA has the most meaningful effects. Specifically, high levels of TA provision were not consistently associated with better outcomes in the current investigation. In fact, high-level users of TA services tended to be those who were struggling to meet at least some of their grant goals. Such findings suggest that the relationship between the amount of TA provided and impact is complex and is likely mediated by the level of need of the TA recipient.

With respect to how well TA has been received, grantees indicated an encouragingly high level of satisfaction with TA efforts. Grantees also reported a positive impact of TA on two of the five measured implementation-related outcomes. Regarding alignment between TA offered and TA needed, NTTAC appears to have been successful in matching its efforts with the specific needs of funded SOC sites. Grantees appear to be appropriately seeking out TA in needed areas and NTTAC appears to be resourced adequately to meet this need. Such alignment is a core component of TA identified in previous literature.^8, 9, 21^

Although overall alignment was found to be considerable, the analyses suggest that NTTAC TA consultants may have provided some areas of TA less extensively than might be expected based on grant goals. For example, the topics of trauma-informed systems and supports, evidence-based practice (EBP) implementation, communications, cultural competence, workforce development, service array and access, and SOC infrastructure and governance were reported at a rate lower than grantees listed these topics as goals. However, the degree of under-provision varies widely across topics, and NTTAC TA consultants regularly refer questions on certain topics, such as trauma and communication, to external experts, such as the National Child Traumatic Stress Network.^30^ As such, while NTTAC TA consultants might not directly address these topics, they may be supporting CMHI grantees through referrals to other sources. Furthermore, the finding related to EBP is likely an artifact of the changes made to the TARS reporting system in which TA providers were unable to specifically choose EBP as a TA topic after March 2018.

Finally, the findings suggest that CMHI grantees are demonstrating some movement toward implementing structures designed to support systems of care over time, as measured by the SAIS, particularly in the areas of management and CQI. Although the current data do not link TA efforts to these specific implementation outcomes, the tailored nature of the support provided by NTTAC along with the self-reported impact data suggest that the TA efforts may have at least contributed to site-level outcomes. Given the self-report nature of these data, such conclusions should be considered preliminary. Future analyses are planned to examine the degree to which NTTAC technical support is specifically linked to both systems- and organization-level outcomes.

Together, the findings of this study provide preliminary evidence that NTTAC has created a TA system that reaches its target audience, is responsive to stakeholders needs, has been well-received by TA recipients, and has resulted in some degree of impact on implementation outcomes. These results underscore the importance of considering context when examining relations among TA support and outcomes. As noted above, the relation between TA dosage and outcomes appears to be influenced by factors such as level of need and stage of implementation. Previous research has also emphasized the importance of considering the degree to which TA support is offered in a way that is proactive, timely, and tailored to unique stakeholder needs, strengths, and resources.^5, 8, 21^ These characteristics of effective TA underscore the importance of considering various “outer setting” variables that commonly influence implementation outcomes. For example, the political environment, economic trends, funding resources, and connections across government and non-government agencies influence implementation, and also should be considered by TA providers as they tailor their efforts to support implementation efforts.^31, 32^ Given NTTAC’s focus on supporting state- and local-level implementation and adoption of SOC principles and procedures, such focus on contextual factors is a central component of their TA efforts.

### Limitations

As with any study, there are several limitations associated with the current methods that should be considered before drawing implications from these findings. First, the TARS came online in July 2014, so the first year of TA provided to 2013 grantees is not captured in the current TARS-based analyses. This impacted the ability to fully assess patterns of TA usage and its possible impact on goal implementation over time. Performing similar analyses for the 2015/2017 grantee cohort, when data are available, would help overcome this limitation.

Limitations of TARS may have influenced the level of alignment between TA provided and grantee needs. For example, TARS options for reporting categories of TA topics have changed over time. Although attempts were made to accurately categorize TA topics for the purposes of the current study, it is possible that some TA topics are either over- or under-represented based on coding errors. As noted earlier in this article, research assistants worked to establish perfect agreement on coding categories; however, the lack of an objective measure of inter-rater reliability is a limitation of this study. Furthermore, while grantee goals served as a proxy for TA needs in this study, it is likely that grantee needs are not limited to those expressed within their original set of goals. Over the course of a grant cycle, it is likely that needs will evolve, with original needs being met and new ones emerging. As such, the data provided on alignment between TA and grantee needs in the current study represents a point-in-time estimate that may not fully capture the complexity of the processes through which TA providers work to meet a dynamic set of needs.

In addition, the degree to which TA providers entered activities into TARS varied. Administrators from NTTAC report that missing data may mean that TA dosage estimates are somewhat lower than actually provided. There also is variation in the way some providers summarize their TA contacts. Specifically, TA consultants for tribal grantees tended to summarize TA activities on a monthly basis, and some of these hours included day-long trainings in addition to individualized TA sessions, which results in higher TA dosage estimates for these providers as compared with others. These data entry practices may help explain the high rates of TA reported for tribal grantees.

An overarching limitation in these efforts to document the impact of TA provided by NTTAC is the lack of a clear assessment of implementation success. Although the SAIS provides some indicator of sites’ success, it relies on self-report from a relatively small number of informants. Moreover, due to our need to rely on existing data from the national evaluation, only one SAIS data point per site was available for the current analysis, limiting the ability to document improvement over time and its association with TA provided.

Finally, the lessons learned from this study are constrained by limitations associated with evaluating TA in the “real world.” As noted above, TA outcomes are influenced by a host of mitigating circumstances, such as changes in political leadership, economic trends, and emergent crises. As such, it is difficult to measure causal relations between TA and system or service improvements. In the current study, observed outcomes, including perceptions, attitudes, and systems-level changes, may have been influenced by a variety of contextual factors.

## Implications for Behavioral Health

A range of empirical studies as well as observations from veteran researchers and policy-makers point to low and declining rates of state and federal funding for both support to and research on implementation support for effective behavioral health practices.^33–35^ As such, it is critical that established providers of TA such as NTTAC have guidance to efficiently and effectively achieve their mission. Unfortunately, the literature focused on how best to provide evidence-based TA is even more limited than that focused on specific evidence-based behavioral health treatments.

However, the current findings have several implications for TA efforts designed to support behavioral health systems. Specifically, the results summarized above build on and reinforce the emerging research base focused on evidence-based TA,^9, 21^ and suggest that factors such as matching TA content and dosage to the individual needs of TA recipients may contribute to successful TA provision that ultimately promotes improved behavioral health systems, services, and treatments. Moreover, the current results suggest that NTTAC’s focus on systems-level changes may be linked to progress on SOC implementation. Such findings underscore the importance of attending to outer setting factors when designing and implementing TA efforts aimed at supporting the implementation of behavioral health services and systems.^31^

Furthermore, the findings of the current study are consistent with the work of Le and colleagues, who suggest that TA providers must consider how systems-level outer setting factors interact with inner setting characteristics such as available resources, prioritization of goals, group culture, and organizational readiness to impact the unique needs of different members of the behavioral health workforce.^5, 6, 9, 31, 32^ An awareness of these and other contextual factors may help TA providers understand strengths and constraints of the service delivery systems in which TA recipients are embedded, and this will allow them to tailor TA efforts accordingly.^32^ Within the field of behavioral health, TA providers should attend to the intersection of inner and outer setting variables that ultimately impact implementation. By carefully considering the degree to which TA recipients are influenced by constraints and opportunities both within and outside of their organizations, TA providers will be better able to tailor their efforts and promote advances in behavioral health services and systems.

The next step in the evolution of evidence-based TA is to test the degree to which these components are linked to implementation success. The authors of this article are currently collecting data in a mixed methods study designed to assess the degree to which this is the case among CMHI grantees. As this research base continues to grow, TA approaches can evolve to reflect the core components that emerge. Given the recent growth of TA efforts and the financial expenditures to help support it, one could argue that stakeholders in the field of children’s behavioral health deserve no less than an ongoing, concerted effort to evaluate its impact. Such efforts will help improve TA quality and impact and will ultimately have a positive effect on the behavioral health outcomes of the children, youth, and families who receive support from public child serving systems.
